# Targeted Disruption of Melanin Biosynthesis Genes in the Human Pathogenic Fungus *Lomentospora prolificans* and Its Consequences for Pathogen Survival

**DOI:** 10.3390/ijms17040444

**Published:** 2016-03-24

**Authors:** Ayat Al-Laaeiby, Michael J. Kershaw, Tina J. Penn, Christopher R. Thornton

**Affiliations:** 1 Biosciences, College of Life and Environmental Sciences, University of Exeter, Stocker Road, Exeter EX4 4QD, UK; aiea201@exeter.ac.uk (A.A.-L.); M.J.Kershaw@exeter.ac.uk (M.J.K.); T.J.Penn@exeter.ac.uk (T.J.P.); 2 Cell and Biotechnology Research Unit, College of Science, University of Basrah, Basrah 61004, Iraq

**Keywords:** *Lomentospora*, melanin, fungus, pathogen, scytalone dehydratase, polyketide synthase, tetrahydroxynaphthalene reductase, gene

## Abstract

The dematiaceous (melanised) fungus *Lomentospora* (*Scedosporium*) *prolificans* is a life-threatening opportunistic pathogen of immunocompromised humans, resistant to anti-fungal drugs. Melanin has been shown to protect human pathogenic fungi against antifungal drugs, oxidative killing and environmental stresses. To determine the protective role of melanin in *L. prolificans* to oxidative killing (H_2_O_2_), UV radiation and the polyene anti-fungal drug amphotericin B, targeted gene disruption was used to generate mutants of the pathogen lacking the dihydroxynaphthalene (DHN)-melanin biosynthetic enzymes polyketide synthase (PKS1), tetrahydroxynapthalene reductase (4HNR) and scytalone dehydratase (SCD1). Infectious propagules (spores) of the wild-type strain 3.1 were black/brown, whereas spores of the PKS-deficient mutant Δ*Lppks1*::*hph* were white. Complementation of the albino mutant Δ*Lppks1*::*hph* restored the black-brown spore pigmentation, while the 4HNR-deficient mutant Δ*Lp4hnr*::*hph* and SCD-deficient mutant Δ*Lpscd1*::*hph* both produced orange-yellow spores. The mutants Δ*Lppks1*::*hph* and Δ*Lp4hnr*::*hph* showed significant reductions in spore survival following H_2_O_2_ treatment, while spores of Δ*Lpscd1*::*hph* and the Δ*Lppks1*::*hph* complemented strain Δ*Lppks1*::*hph*:PKS showed spore survivals similar to strain 3.1. Spores of the mutants Δ*Lp4hnr*::*hph* and Δ*Lpscd1*::*hph* and complemented strain Δ*Lppks1*::*hph*:PKS showed spore survivals similar to 3.1 following exposure to UV radiation, but survival of Δ*Lppks1*::*hph* spores was significantly reduced compared to the wild-type strain. Strain 3.1 and mutants Δ*Lp4hnr*::*hph* and Δ*Lppks1*::*hph*:PKS were resistant to amphotericin B while, paradoxically, the PKS1- and SCD1-deficient mutants showed significant increases in growth in the presence of the antifungal drug. Taken together, these results show that while melanin plays a protective role in the survival of the pathogen to oxidative killing and UV radiation, melanin does not contribute to its resistance to amphotericin B.

## 1. Introduction

*Lomentospora prolificans* (formally *Scedosporium prolificans*) is a dematiaceous (melanised) fungus that has emerged over recent years as a serious and often life-threatening pathogen of immunocompromised individuals including those with AIDS, haematological malignancies and bone marrow and solid organ transplants [1,2,3,4,5,6,7,8]. In neutropenic patients, deep-seated infections disseminate rapidly with associated mortality rates of up to 80% [1,9]. Infections due to near-drowning were reported by the World Health Organisation after the Great Eastern Japan earthquake and tsunami in 2011 [10,11]. Recovery of *L. prolificans* from the sputum of cystic fibrosis patients has also been reported, but disease exacerbation due to the fungus has yet to be established [12].

A characteristic of *L. prolificans* and other dematiaceous human pathogenic fungi, which accounts for high patient mortalities, is their widespread resistance to systemic anti-fungal drugs currently available in the clinic including the broad-spectrum polyene macrolide amphotericin B, the first line therapy for a number of invasive mycoses [13,14,15,16,17,18]. *In vitro* drug susceptibilities of these melanised fungi are typically lower than for non-melanised fungi and the protective role of melanin in antifungal drug resistance and to environmental stresses has been advocated [19,20,21,22,23]. Melanin has been shown to protect fungal pathogens against the antifungal drug amphotericin B [19,24,25], oxidative killing of infectious propagules [22,26,27,28,29], destruction of propagules by phagocytic cells of the immune system [22,25,30], antimicrobial peptides [31] and can confer tolerance against UV, solar and gamma radiations [25,32,33]. Furthermore, melanin has been shown to be an important virulence factor in both plant and human pathogenic fungi [22,25,28,34,35].

**Figure 1:**
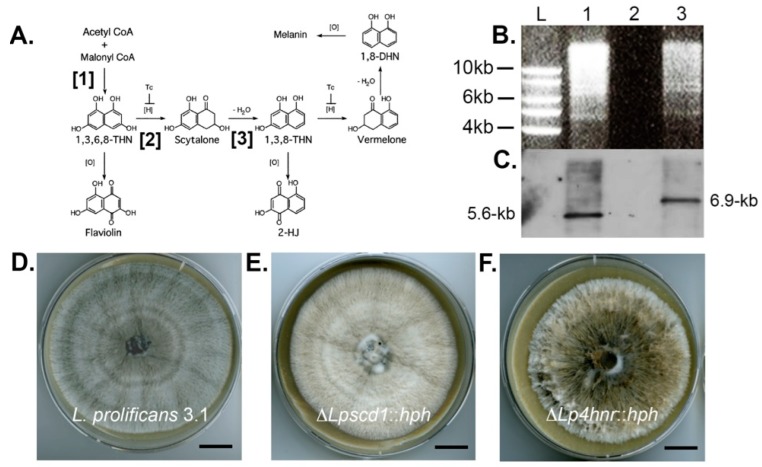

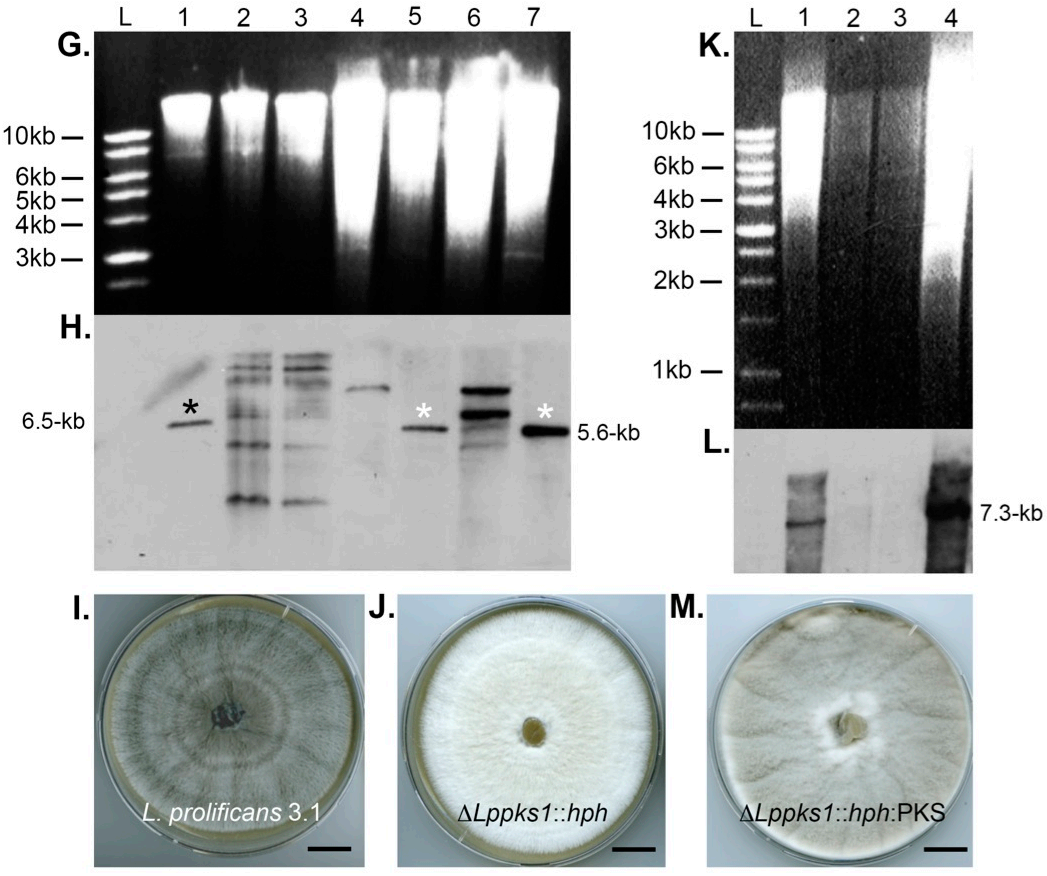
Southern blot analysis of targeted scytalone dehydratase 1 (*SCD1*) and polyketide synthase (*PKS1*) gene replacements and *PKS1* complementation and resultant phenotypes of Δ*Lpscd1*::*hph*, Δ*Lppks1*::*hph* and Δ*Lppks1*::*hph*:PKS mutants. (**A**) Schematic diagram showing the three different steps of dihydroxynaphthalene (DHN)-melanin biosynthesis disrupted by targeted gene deletion in this study. Targeted disruption of *PKS1* (step [1]) prevents production of the melanin precursor 1,3,6,8-tetrahydroxynaphthalene (1,3,6,8-THN), disruption of tetrahydroxynapthalene reductase (*4HNR*) (step [2]) prevents reduction of 1,3,6,8-THN to scytalone and disruption of *SCD1* (step [3]) prevents dehydration of scytalone to 1,3,8-THN; (**B**) Genomic DNA of the *L. prolificans* wild-type strain 3.1 (lane 1) and the putative transformant Δ*Lpscd1*::*hph* (lane 3) were digested with the restriction enzyme *Bg*lII, fractionated by gel electrophoresis and blotted onto Hybond-NX membrane; (**C**) Lane L contains DNA size marker and lane 2 was left blank. The membrane was probed with a 1.0-kb fragment upstream of the *SCD1* ORF. The presence of the single 6.9-kb band in lane 3, compared to the single 5.7-kb band in lane 1, indicates successful replacement of the *SCD1* gene; (**D**) Colony morphology of strain 3.1 after 2-week growth on oatmeal agar (OA) at 30 °C showing typical grey phenotype; (**E**) Morphology of mutant Δ*Lpscd1*::*hph* after 2-weeks growth on OA at 30 °C showing abnormal beige pigmentation and comparison to the yellow-grey tetrahydroxynaphthalene reductase-deficient mutant Δ*Lp4hnr*::*hph* (**F**) developed previously [38]. Scales bars in **D**–**F** = 1.5 cm; (**G**) Genomic DNA of wild-type strain 3.1 (lane 1) and putative Δ*Lppks1*::*hph* transformants (lanes 2 to 7) were digested with the restriction enzyme *Hpa*I, fractionated by gel electrophoresis and blotted onto Hybond-NX membrane. Lane L contains DNA size marker; (**H**) The membrane was probed with a 1.9-kb right flank fragment of the *PKS1* ORF. The presence of single 5.6-kb bands in lanes 5 and 7 (indicated by white asterisks), compared to the single 6.5-kb band in lane 1 (indicated by black asterisk), indicates successful replacement of the *PKS1* gene in these two strains; (**I**) Colony morphology of wild-type strain 3.1 after 2-weeks growth on OA at 30 °C showing typical grey phenotype; (**J**) Morphology of the putative Δ*Lppks*::*hph* mutant corresponding to lane 7 of the Southern blot shown in panel **H**, after 2-weeks growth on OA at 30 °C. Note the albino phenotype and complete loss of pigmentation. Scales bars in **I** and **J** = 1.5 cm; (**K**) Genomic DNA of strain 3.1 (lane 1), the two Δ*Lppks1*::*hph* mutants corresponding to lanes 5 and 7 of the Southern blot shown in panel **H** (lanes 2 and 3) and a putative Δ*Lppks1*::*hph*:PKS complemented strain (lane 4) derived from the Δ*Lppks1*::*hph* mutant shown in lane 7 of the Southern blot in panel **H**. DNA was digested with the restriction enzyme *Bg*lII, fractionated by gel electrophoresis and blotted onto Hybond-NX membrane; (**L**) Lane L is DNA size marker. The membrane was probed with a 0.8-kb fragment of the *PKS1* ORF. The presence of a single 7.3-kb band in lane 4 (comparable to lane 1) indicates successful complementation of the Δ*Lppks1*::*hph* mutant and production of the complemented strain Δ*Lppks1*::*hph*:PKS; and (**M**) Colony morphology of the Δ*Lppks1*::*hph* complemented strain Δ*Lppks1*::*hph*:PKS after 2-weeks growth on OA at 30 °C, showing restoration of melanin production and a grey phenotype similar to that of the wild-type strain 3.1 (**D** and **I**). Scale bar = 1.5 cm.

As with many other dematiaceous fungi, *Lomentospora prolificans* produces the polymer dihydroxynaphthalene (DHN)-melanin via a biosynthetic pathway (Figure 1A) that starts with the precursor malonyl-CoA [22,36,37]. The first step (Figure 1A, step [1]) in the pathway is catalysed by the enzyme polyketide synthase (PKS1), which converts malonyl-CoA to 1,3,6,8-tetrahydroxynaphthalene (1,3,6,8-THN). 1,3,6,8-THN is reduced by the enzyme tetrahydroxynapthalene reductase (4HNR) to scytalone (Figure 1A step [2]). Scytalone is then dehydrated enzymatically by scytalone dehydratase (SCD1) to 1,3,8-trihydroxynaphthalene (Figure 1A, step [3]), which is in turn reduced, possibly by a second reductase, to vermelone. A further dehydration step, possibly also catalysed by SCD1, leads to the intermediate 1,8-DHN. Subsequent steps are thought to involve dimerization of the 1,8-DHN molecules followed by polymerisation.

In a previous study [38], we reported the generation of a monoclonal antibody specific to *L. prolificans* and identified its antigen through mass spectrometry and targeted gene disruption as the melanin biosynthetic enzyme 1,3,6,8-tetrahydroxynapthalene reductase [38]. As an extension to this study, we set out here to determine the role of this enzyme and two other enzymes involved in DHN-melanin production (scytalone dehydratase and polyketide synthase) in melanisation of *L. prolificans* and to investigate the consequences of enzyme loss for the survival of the pathogen. To this end, we use targeted disruption of polyketide synthase and scytalone dehydratase-encoding genes to generate enzyme-deficient mutants of the fungus and determine their sensitivities and that of the tetrahydroxynapthalene reductase-deficient mutant [38] to stresses incurred by UV radiation, oxidative killing and amphotericin B. Using this strategy, we show that melanin confers tolerance of the fungus to UV and oxidative killing, but does not contribute to its resistance to the polyene anti-fungal drug.

## 2. Results

### 2.1. Targeted Disruption of L. prolificans Melanin Biosynthesis Genes and Recovery of Putative Mutants

In this study, we used targeted gene replacement to disrupt production of the enzymes polyketide synthase (PKS) and scytalone dehydratase (SCD) that catalyse steps [1] and [3] of DHN-melanin biosynthesis in *L. prolificans* as shown in Figure 1A. Production of a mutant strain (Δ*Lp4hnr*::*hph*) deficient in the enzyme 1,3,6,8-tetrahydroxynapthalene reductase that catalyses step [2] of melanin biosynthesis in the fungus (Figure 1A) was reported recently [38] and is included in this study for comparison to the newly developed mutants. Targeted gene disruptions were carried out following the split marker method [39]. For targeted replacement of the SCD-encoding gene *SCD1*, two rounds of PCR were required to complete the process. In the first round PCR, the left (upstream) (1.0-kb) and right (downstream) (0.9-kb) flanking regions of the *SCD1* ORF were amplified using primer pairs Lpscd1-LFF/Lpscd1-LFR and Lpscd1-RFF/Lpscd1-RFR designed to include an extension complementary to the hygromycin phosphotransferase gene (*HPH*) conferring resistance to the antibiotic hygromycin B (Table 1). The left and right fragments of the *HPH* gene were amplified from pCB1004 with primer pairs HY split/M13F and M13R/YG split, respectively. In a second round of PCR, the left flank was fused with one half of the hygromycin cassette (HY) (1.2-kb) and the right flank with the other half (YG) (0.8-kb). The second round PCR products LF+HY (2.1-kb) and RF+YG (1.7-kb) were used for protoplast transformation. Homologous recombination resulted in the replacement of the *SCD1* ORF with the functional *HPH* gene. Putative Δ*Lpscd1*::*hph* mutants were selected based on resistance to the antibiotic hygromycin B (600 µg/mL).

Targeted replacement of the *PKS1* gene was performed using a similar experimental strategy. Primer pairs Lppks1-LFF/Lppks1-LFR and Lppks1-RFF/Lppks1-RFR were used for amplification of 1.9-kb fragments from 5′ and 3′ flanks of the ORF respectively. The left and right fragments of the *HPH* gene were amplified as described. In a second round of PCR, the left flank was fused with one half of hygromycin cassette (HY) (1.2-kb) and the right flank with the other half of the hygromycin cassette (YG) (0.8-kb). The second round PCR products LF+HY (3.1-kb) and RF+YG (2.7-kb) were used for protoplast transformation. Homologous recombination resulted in the replacement of the *PKS1* ORF with the functional *HPH* gene. Putative Δ*Lppks1*::*hph* mutants were similarly selected based on resistance to 600 µg/mL of the antibiotic hygromycin B.

### 2.2. Confirmation of Gene Disruptions by Southern Blotting and Abnormal Melanisation

Hygromycin B resistant mutants were investigated further by Southern blot analysis. A single putative Δ*Lpscd1*::*hph* mutant and six Δ*Lppks1*::*hph* putative mutants were analysed (Figure 1). Genomic DNA from the putative Δ*Lpscd1*::*hph* mutant and the wild-type strain 3.1 were digested with the restriction enzyme *Bg*lII and the enzyme *Hpa*I for putative Δ*Lppks1*::*hph* transformants and wild-type strain 3.1 control. The products were separated by agarose gel electrophoresis (Figure 1B,G, respectively) and blotted onto Hybond-NX membranes (Figure 1C,H, respectively). Respective membranes were probed with a 1.0-kb fragment upstream of the *SCD1* ORF (Figure 1C) or a 1.9-kb right flank fragment of the *PKS1* ORF (Figure 1H). The single putative Δ*Lpscd1*::*hph* mutant (Figure 1C, lane 3) showed the correct fragment size of 6.9-kb compared to the 5.6-kb fragment of the wild-type strain (Figure 1C, lane 1), while two Δ*Lppks1*::*hph* mutants (Figure 1H, lanes 5 and 7 indicated by white asterisks) were identified with the correct fragment size of 5.6-kb compared to the 6.5-kb fragment of the wild-type strain (Figure 1H, lane 1 indicated by black asterisk). Targeted disruption of the melanin biosynthesis gene *SCD1* encoding the melanin biosynthesis enzyme scytalone dehydratase and *PKS1* encoding the enzyme polyketide synthase resulted in abnormal pigmentation in the Δ*Lpscd1*::*hph* and Δ*Lppks1*::*hph* mutants when compared to the wild-type strain 3.1. The wild-type 3.1 strain is grey (Figure 1D,I) in contrast to the beige colour of the Δ*Lpscd1*::*hph* mutant (Figure 1E), while the tetrahydroxynaphthalene reductase-deficient mutant Δ*Lp4hnr1*::*hph* mutant (Figure 1F), generated in a previous study [38], is yellow-grey. The two Δ*Lppks1*::*hph* mutants confirmed in Southern blots (Figure 1H, lanes 5 and 7) were albino (white), exhibiting complete loss of pigmentation (Figure 1J; the Δ*Lppks1*::*hph* mutant corresponding to lane 7 is panel H is shown) compared to the grey wild-type strain 3.1.

### 2.3. Complementation of the ΔLppks1::hph Mutant and Restoration of Melanin Production

An albino Δ*Lppks1*::*hph* mutant (Figure 1H, lane 7) was complemented by integration of a DNA fragment consisting of 3.0-kb of promoter region, 6.7-kb of the ORF and 0.6-kb terminator region of the *PKS1* gene to restore gene functionality and concomitant melanin biosynthesis. The *PKS1* fragment was amplified by PCR using the gene-specific primers Lppks-f1 and Lp-pksr1 (Table 1), designed to include 15-bp of sequence homologous to the ends of the linearized vector pCB1532. The vector pCB1532, which contains the sulfonylurea resistance allele of the *Magnaporthe oryzae* *ILV1* gene [40], was linearized by digestion with the restriction enzymes *Bam*HI and *Hind*III. The PCR product was ligated to linearized pCB1532 vector and used to transform competent *E. coli* cells, which were selected based on white-blue screening. Plasmid DNA was purified and sequenced to confirm the correct *PKS1* insertion and the vector then transformed into the Δ*Lppks1*::*hph* mutant. Putative Δ*Lppks1*::*hph*:PKS complementation mutants were selected based on resistance to sulfonylurea. Genomic DNA of wild-type 3.1, Δ*Lppks1*::*hph* and putative complemented mutants was digested with the restriction enzyme *Bg*lII, fractionated by gel electrophoresis (Figure 1K) and blotted onto a Hybond-NX membrane (Figure 1L). The membrane was probed with a 0.8-kb fragment of the *PKS1* ORF. The probe hybridized to a 7.3-kb fragment present in the wild-type 3.1 and complemented strains (Figure 1L, lanes 1 and 4), which was absent in the two Δ*Lppks1*::*hph* mutants (Figure 1L, lanes 2 and 3). Complementation of the polyketide synthase-deficient mutant restored melanin production (Figure 1M), resulting in a grey phenotype in the Δ*Lppks1*::*hph*:PKS complementation mutant similar to the wild-type strain 3.1 (Figure 1D,I).

### 2.4. Growth, Sporulation and Spore Pigmentation

**Figure 2:**
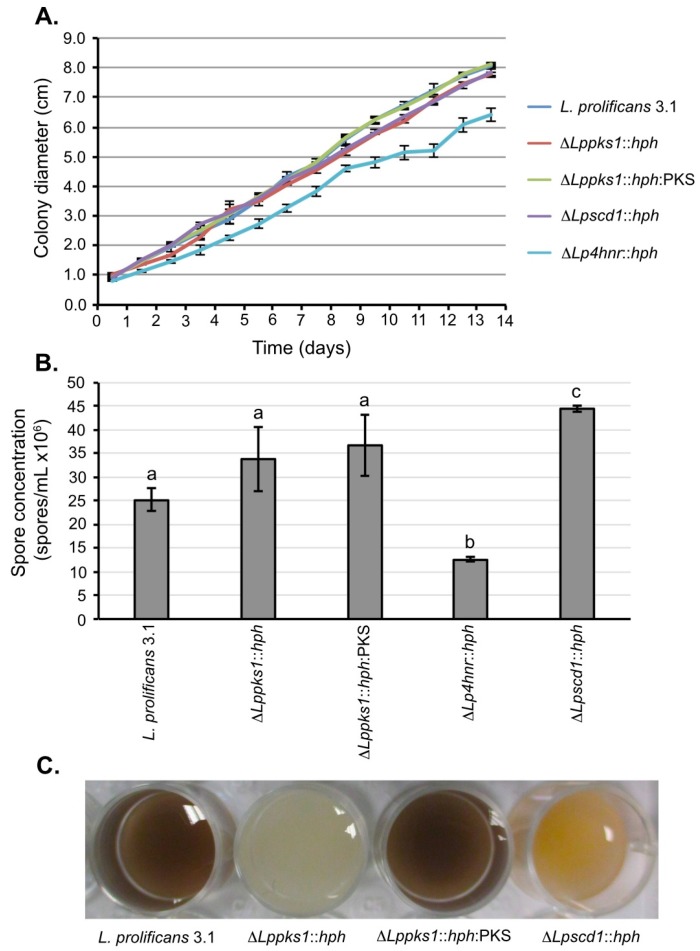
Phenotypic characterization of *L. prolificans* 3.1 and melanin mutants. (**A**) Colony diameters of fungi grown on OA for 14 days. By day 14, the mutant Δ*Lp4hnr*::*hph* had significantly reduced growth when compared to the wild-type strain (*p* < 0.05; Student’s *t*-test). All other mutants showed growth comparable to strain 3.1 over the 14 day period. Each point is the mean of 3 replicates ± standard error; (**B**) Quantification of spore concentrations from 14-day-old OA plate cultures. Spore concentration of the mutant Δ*Lp4hnr*::*hph* was significantly reduced (*p* < 0.05, analysis of variance (ANOVA)) when compared to strain 3.1 and the other mutants, whereas spore concentration was significantly increased in the mutant Δ*Lpscd1*::*hph* when compared to the other strains. There were no significant differences in the spore concentrations of strains 3.1, Δ*Lppks1*::*hph* and Δ*Lppks1*::*hph*:PKS. Each bar is the mean of 3 replicates ± standard error and bars with similar letters indicate that differences in means are not statistically significant at *p* < 0.05 (ANOVA); (**C**) Colors of spore suspensions of *L. prolificans* 3.1 and the mutants Δ*Lppks1*::*hph*, Δ*Lppks1*::*hph*:PKS and Δ*Lpscd1*::*hph*.

Growth of the fungi on OA revealed no significant differences in growth rates compared to strain 3.1, with the exception of mutant Δ*Sp4hnr*::*hph*. With this mutant, there was a significant reduction in colony diameter by day 14 compared to the wild-type strain (Figure 2A), consistent with previous findings [38]. While spore production in the 14-day-old cultures was not significantly different between strain 3.1 and mutants Δ*Lppks1*::*hph* and Δ*Lppks1*::*hph*:PKS, production was significantly reduced in the mutant Δ*Lp4hnr*::*hph* and significantly increased in the mutant Δ*Lpscd1*::*hph* when compared to the wild-type strain (Figure 2B). Spore suspensions prepared from the 14-day-old cultures showed marked differences in pigmentations. Spore suspensions from strain 3.1 were black-brown, whereas those from the albino mutant Δ*Lppks1*::*hph* were white (Figure 2C). Spores of the complemented strain Δ*Lppks1*::*hph*:PKS were black-brown, showing that complementation had restored melanin biosynthesis resulting in a similar spore pigmentation to that of the wild-type strain. The mutant Δ*Lpscd1*::*hph* produced orange-yellow spore suspensions similar to that of the Δ*Lp4hnr*::*hph* generated previously [38].

### 2.5. Sensitivities to H_2_O_2_

**Figure 3:**
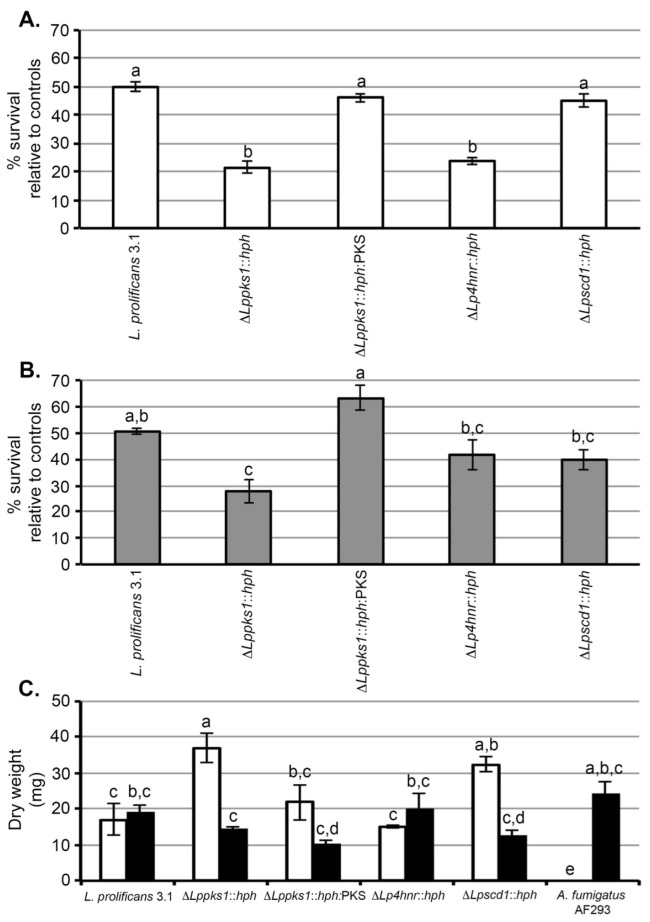
Sensitivities of *L. prolificans* 3.1 and melanin mutants to UV, H_2_O_2_ and the antifungal drug amphotericin B. (**A**) Sensitivities of spores to oxidative killing by H_2_O_2_. Spores were exposed to 160 mM H_2_O_2_ or phosphate buffer only (control) for 60 min and then plated onto SDA. After incubation in the dark for 3 day at 30 °C, the number of colonies derived from germinated spores were counted and the % survival for each strain determined relative to matched controls (untreated spores). Each bar is the mean of 3 replicates ± standard error and bars with similar letters indicate that differences in means are not statistically significant at *p* < 0.05 (ANOVA). Under these conditions, ~50% of treated spores of the wild-type strain 3.1 survived compared to matched control spores. Compared to strain 3.1, there was a significant reduction in % survival of mutants Δ*Lppks1*::*hph* and Δ*Lp4hnr*::*hph* due to exposure to H_2_O_2_. There was no significant effect of H_2_O_2_ on the survival of mutant Δ*Lpscd1*::*hph* or on the survival of Δ*Lppks1*::*hph*:PKS compared to 3.1, showing that complementation of mutant Δ*Lppks1*::*hph* had restored spore survival to wild-type levels; (**B**) Sensitivities of spores to UV light. Spores were exposed to a UV dose of 200 mJ/cm^2^ and then plated onto SDA. After incubation in the dark for 3 days at 30 °C, the number of colonies derived from germinated spores were counted and the % survival for each strain determined relative to matched controls (untreated spores). Each bar is the mean of 3 replicates ± standard error and bars with similar letters indicate that differences in means are not statistically significant at *p* < 0.05 (ANOVA). Under these conditions, ~50% of treated spores of the wild-type strain 3.1 survived compared to matched control spores. Compared to strain 3.1, there was a significant reduction in % survival of mutant Δ*Lppks1*::*hph* due to exposure to UV. There was no significant effect of UV exposure on the survival of mutants Δ*Lpscd1*::*hph* and Δ*Lp4hnr*::*hph*. Similarly, there was no significant difference in the % survival of Δ*Lppks1*::*hph*:PKS spores compared to 3.1, showing that complementation of mutant Δ*Lppks1*::*hph* had restored spore survival to wild-type levels; (**C**) Sensitivities of strains to the polyene antifungal drug amphotericin B. Each bar is the mean of 3 replicates ± standard error and bars with similar letters indicate that differences in means are not statistically significant at *p* < 0.05 (ANOVA). The black bars are the controls lacking amphotericin B and the open bars show growth in the presence of the drug. Growth (dry weight in mg) of the drug-resistant wild-type strain 3.1 and mutant Δ*Lp4hnr1*::*hph* was unaffected by the drug. Similarly, there was no significant difference in the growth of the complemented strain Δ*Lppks1*::*hph*:PKS in the presence and absence of the drug, showing that restoration of melanin biosynthesis re-established drug insensitivity of the fungus to wild-type levels. In contrast, significant increases in growth were found in the albino mutant Δ*Lppks1*::*hph* and mutant Δ*Lpscd1*::*hph* in the presence of the drug, while the drug completely inhibited growth of the control pathogen *Aspergillus fumigatus.*

Exposure of spores of strain 3.1 to 160 mM H_2_O_2_ for 60 min resulted in 50% survival relative to its matched control (non-treated spores) (Figure 3A). When compared to the wild-type strain, the same treatment resulted in significant (*p* < 0.05) reductions in survival of the mutants Δ*Lppks1*::*hph* and Δ*Lp4hnr*::*hph*, with survival percentages relative to matched controls of 21% and 24% respectively. There was no significant difference in the effect of H_2_O_2_ on survival of Δ*Lppscd1*::*hph* spores when compared to strain 3.1 with a percentage survival, relative to its matched control, of 45%. A similar result was found with the mutant Δ*Lppks1*::*hph*:PKS, with a percentage survival relative to its matched control of 46%, indicating that complementation had restored survival to wild-type levels.

### 2.6. Sensitivities to UV Radiation

A UV dose of 200 mJ/cm^2^ resulted in 51% survival of strain 3.1 spores relative to non-irradiated control spores (Figure 3B). Under the same treatment conditions, there was a significant (*p* < 0.05) decrease in percentage survival of the mutant Δ*Lppks1*::*hph* compared to the wild-type, with a percentage survival of 28%. There was no significant difference in effect of UV irradiation on the percentage survival of the mutants Δ*Lpscd1*::*hph* and Δ*Lp4hnr*::*hph* when compared to strain 3.1, with percentages of 40% and 42% respectively. A similar result was found with the mutant Δ*Lppks1*::*hph*:PKS, with a percentage survival relative to its matched control of 63%, indicating that complementation had restored survival to wild-type levels.

### 2.7. Sensitivities to Amphotericin B

All strains of *L. prolificans* tested to date are resistant to the broad-spectrum antifungal drug amphotericin B [14,15]. Here, we tested the susceptibility of the wild-type strain 3.1 and the melanin biosynthesis mutants to the drug and included, as a positive control, the drug-sensitive pathogen *Aspergillus fumigatus* (AF293). We chose a concentration of 32 µg/mL, having first established that strain 3.1 of the fungus is resistant to concentrations >16 µg/mL, consistent with other studies [13,14,15,16,17,18]. Growth (measured as dry weight of mycelium in mg) of the drug-resistant wild-type strain 3.1 and mutant Δ*Lp4hnr1*::*hph* was unaffected by the drug. Similarly, there was no significant difference in the growth of the complemented strain Δ*Lppks1*::*hph*:PKS in the presence and absence of the drug, showing that restoration of melanin biosynthesis re-established drug insensitivity of the fungus to wild-type levels. Unexpectedly, significant increases in growth were found in the albino mutant Δ*Lppks1*::*hph* and mutant Δ*Lpscd1*::*hph* in the presence of the drug, while the drug completely inhibited growth of the control pathogen *Aspergillus fumigatus* as expected.

## 3. Discussion

*Lomentospora prolificans* is a dematiaceous (melanised) fungus that causes life-threatening disseminated infections in immunocompromised humans and patients with underlying respiratory problems [1,2,3,4,5,6,7,8,9,10,11,12]. Disseminated infections known as scedosporiosis are caused by infectious propagules (spores) of the fungus that are inhaled, germinate and infect the lungs due to impaired respiratory immunity including loss of oxidative killing by macrophages and neutrophils. The pathogen displays inherent resistance to many of the mold-active antifungal drugs used in the clinical setting including the polyene amphotericin B [13,14,15,16,17,18]. The pigment melanin is an integral component of the fungal cell wall conferring protection to stresses that involve cell damage such as UV radiation and reactive oxygen species [22,25,26,27,28,29,30,32,33] and which has been shown in other human pathogenic fungi to contribute to amphotericin B resistance [24,25,41]. In this study, we set out to investigate the role of melanin in resistance of *L. prolificans* to UV radiation, to oxidative killing by H_2_O_2_, and inhibition by amphotericin B. To do this, we used targeted disruption of melanin biosynthesis genes to generate mutants blocked at different stages in the DHN-melanin pathway.

In a previous study [38], we used targeted gene disruption to generate an *L. prolificans* mutant (Δ*Lp4hnr*::*hph*) lacking the DHN-melanin enzyme tetrahydroxynaphthalene reductase (4HNR) which catalyzes the reduction of 1,3,6,8-THN to scytalone (Figure 1A, step [2]). In this study, we used a similar process to generate additional mutants, Δ*Lppks1*::*hph* and Δ*Lpscd1*::*hph*, that lack polyketide synthase (PKS1) and scytalone dehydratase (SCD1) enzyme activities, respectively. Furthermore, we complemented the PKS-deficient mutant to restore melanin biosynthesis in the Δ*Lppks1*::*hph* mutant. The split marker technique of homologous recombination used here enables exchange of the open reading frame (ORF) of the enzyme-encoding gene with the hygromycin B phosphotransferase (*HPH*)-encoding gene, a selectable marker conferring resistance to the antibiotic hygromycin B. The procedure was carried out by amplification and fusion of linear DNA fragments to create the hygromycin-resistance cassette. A previous study had shown that the size of the sequences upstream and downstream of the ORF affects transformation efficiency [42] and so the lengths of flanking fragments used for homologous recombination were designed according to the size of each ORF. For the PKS-encoding gene, the flanks were approximately 1.9-kb (left flank) and 1.9-kb (right flank) owing to the large size of the *PKS* ORF (6.7-kb). In the case of the SCD-encoding gene, the flank sizes were shorter (approximately 1.0-kb and 0.9-kb for left and right flanks respectively), owing to the smaller size of the *SCD* ORF of approximately 0.7-kb.

Using this strategy, we were able to generate enzyme-deficient mutants that had abnormal pigmentation due to disruption of normal DHN-melanin biosynthesis. Previously, Ruiz-Diez and co-workers [43] used UV mutagenesis to generate melanin-deficient (mel^−^) mutants of *L. prolificans* that had unstable white phenotypes that reverted to the olive-grey to black characteristic of the wild-type strain, or mutants that had stable white phenotypes that varied in colony morphologies. Unlike UV mutagenesis, which is a random mutation process, targeted gene disruption allowed us to mutate individual enzyme-encoding genes involved in melanin biosynthesis in a highly specific way and to generate stable enzyme-deficient mutants. In the case of Δ*Lpscd1*::*hph*, colonies of the mutant were beige while its spore suspensions were orange-yellow. A similar spore pigmentation was shown in the Δ*Lp4hnr*::*hph* mutant developed previously that accumulated flaviolin as a shunt product [38]. Wang and co-workers [44] produced reddish-brown mutants lacking the corresponding *SCD1* gene in the ascomycete *Grosmannia clavigera* and showed this color was due to accumulation of the intermediate scytalone. Targeted disruption of the *L. prolificans* PKS-encoding gene resulted in the production of albino (white) Δ*Lppks1*::*hph* mutants, similar to the white PKS-deficient mutants of *Bipolaris oryzae* and *A. fumigatus* developed elsewhere [45,46]. Complementation of the albino Δ*Lppks1*::*hph* mutant was successfully achieved, resulting in a mutant Δ*Lppks1*::*hph*:PKS with grey pigmentation similar to that of the wild-type strain.

Changes to normal melanin production in fungi can alter their morphology. Tseng and co-workers [47] showed that engineering the entomopathogen *Metarhizium anisopliae* to express DHN-melanin biosynthesis genes increased spore germination, hyphal branching and appressorium production. Mutants of the fungus *Grosmannia clavigera* with *PKS* and *SCD* gene disruptions had albino and reddish-brown phenotypes respectively, but had no marked differences in morphology, sporulation, or in vegetative growth compared to the wild-type strain. In the current study, vegetative growth and was significantly reduced in the 4HNR-deficient mutant Δ*Lp4hnr*::*hph* consistent with previous results [38], while growth was unaffected in the other two mutants and in the complemented strain Δ*Lppks1*::*hph*:PKS. Spore production was reduced in Δ*Lp4hnr*::*hph* and increased in Δ*Lpscd1*::*hph.*

Melanin is known to contribute to the stress tolerances of fungi, including both plant and human pathogens, conferring resistance to UV radiation [33] and to oxidation killing [22,26,27,28,29]. Kheder and co-workers [48] showed that spore germination and radial growth was dramatically reduced in non-melanised strains of the plant pathogen *Alternaria alternata* compared to melanised strains as a consequence of UV exposure, while another study demonstrated resistance to UV light of melanised strains of the human pathogen *Sporothrix* *schenckii* compared to a melanin-deficient mutant [49]. In human pathogenic fungi, melanin acts as a free radical scavenger [50] and is associated with increased tolerance to H_2_O_2_ in *Cryptococcus neoformans*, *Aspergillus* spp., *S. schenckii* and *Fonsecaea pedrosoi* [26,27,28,29,30]. In this study, the albino Δ*Lppks1*::*hph* mutant showed increased sensitivity to UV and H_2_O_2_ in spore survival assays. Restoration of melanisation in the Δ*Lppks1*::*hph*:PKS complemented strain and increased survival of Δ*Lppks1*::*hph*:PKS spores to wild-type levels following UV and H_2_O_2_ exposure, shows that melanin provides protection against UV damage and tolerance to oxidative killing in *L. prolificans*. In contrast, another study knocked down the polyketide synthase gene in *Penicillium marneffei* and found no difference between the wild-type and the mutant in rates of survival following exposure to UV light [51].

The resistance of *L*. *prolificans* to antifungal agents is well documented [13,14,15,16,17,18] and the contribution of melanin to antifungal drug resistance has been investigated in other human pathogenic fungi. Van Duin and co-workers [41] demonstrated the protective role of melanin in *C. neoformans* and *Histoplasma* *capsulatum* against amphotericin B, with non-melanised *C. neoformans* strains showing greater sensitivity to the drug than melanised strains [24]. Contrary to expectations, we found that the albino mutant of *L. prolificans* generated here (Δ*Lppks1*::*hph*) showed significantly increased growth in the presence of amphotericin B, while complementation of the *PKS* gene in the mutant Δ*Lppks1*::*hph*:PKS bestowed insensitivity to the drug, similar to that of the wild-type strain 3.1. The unexpected increase in growth of the albino mutant in the presence of the drug, a phenomenon also found with the Δ*Lpscd1*::*hph* mutant, is difficult to explain. Paradoxical increases in growth of the human pathogens *Candida albicans* and *Aspergillus fumigatus* due to elevated concentrations of the anti-fungal echinocandin drug caspofungin have been reported [52,53], but increased resistance of fungi to a polyene anti-fungal drug as a consequence melanin deficiency has not previously been demonstrated. Indeed, melanin deficient (mel^−^) strains *L. prolificans* generated by UV mutagenesis [43] were shown to be as resistant to amphotericin B, nystatin and azoles as the wild-type parent. Despite this, amphotericin B has been shown to increase the melanin content of melanoma cells [54]. An alternative explanation for the increased growth of mutants Δ*Lppks1*::*hph* and Δ*Lpscd1*::*hph* might be compensation for DHN-melanin loss through alternative pathways of melanin biosynthesis. In addition to DHN-melanins, the ascomycete fungi *Aspergillus fumigatus* and *A. nidulans* are able to synthesize pyomelanin and DOPA-melanin [55]. Similar synthetic capabilities might also be present in *L. prolificans*. Notwithstanding this, we have shown that melanin does not contribute to the resistance of *L. prolificans* to amphotericin B, consistent with the findings of Ruiz-Diez and co-workers [43].

In conclusion, we have shown using targeted deletion of melanin biosynthesis genes that melanin confers *L. prolificans* with protection from oxidative killing by H_2_O_2_ and protection from UV radiation, but is not involved in its resistance to the anti-fungal drug amphotericin B.

## 4. Materials and Methods

### 4.1. Fungal Culture

The *Lomentospora prolificans* wild-type strain 3.1 [38], gene disruption mutants and *Aspergillus fumigatus* strain AF293 were grown routinely on oatmeal agar (OA; O3506, Sigma, Sigma-Aldrich, Poole, Dorset, UK) or Sabouraud dextrose agar (SDA; Sabouraud dextrose broth (SBD; S3306, Sigma) containing 2% agar) at 30 °C under a 16 h fluorescent light regime to induce sporulation. Agar was sterilized by autoclaving at 121 °C for 15 min.

### 4.2. Genomic DNA Extraction

DNA extraction was carried out according to the procedures described in Alastruey-Izquierdo and co-workers [56]. Spores were harvested by flooding OA plate cultures with 20 mL sterile autoclaved Milli-Q water (MQ-H_2_O) and suspension with a sterile L-shaped spreader (Fisher Scientific UK Ltd., Loughborough, Leicestershire, UK). The suspension was filtered through sterile Miracloth (Calbiochem, San Diego, CA, USA) to remove hyphal tissue, centrifuged at 13,000× *g* to pellet spores and the spores re-suspended in sterile MQ-H_2_O. Sterile 75 cm^2^ tissue culture flasks containing 100 mL of tissue culture medium (TCM; RPMI-1640 medium (R0883, Sigma) containing 10% fetal bovine serum (Labtech International Ltd., Uckfield, East Sussex, UK), 2 mM l-glutamine (G7513, Sigma), penicillin and streptomycin) were inoculated with spore suspension to give a final concentration of 10^4^ spores/mL and incubated with shaking (35 rpm) in an Innova 4000 rotary incubator (Eppendorf UK Ltd., Stevenage, Hertfordshire, UK) for 48 h at 30 °C. Hyphal biomass was harvested by filtration through sterile Miracloth, washed with sterile MQ-H_2_O, blotted dry with paper towel (Kimberley-Clark Ltd., West Malling, Kent, UK) and stored at −80 °C until required. Frozen mycelium was ground to a fine powder in liquid N_2_ using a mortar and pestle, the powder transferred to 1.5 mL micro-centrifuge tubes and 800 µL of extraction buffer (0.2 M Tris–HCl, 0.5 M NaCl, 10 mM EDTA, SDS 1%) added followed by 800 µL of phenol:chloroform:isoamyl alcohol mixture (25:24:1). The mixture was shaken gently for 30 s and then centrifuged at 14,000× *g* for 15 min at 4 °C. The upper layer was transferred to a fresh tube and mixed with an equal volume of phenol:chloroform:isoamyl alcohol mixture (25:24:1), vortexed for 30 s and centrifuged as described. The upper layer was combined with an equal volume of chloroform:isoamyl alcohol (24:1) and vortexed for 30 s. Following centrifugation for 5 min at 14,000× *g*, the upper layer was transferred to a fresh tube, the DNA precipitated by adding chilled isopropanol and pelleted by centrifugation at 4 °C for 15 min at 14,000× *g*. The pellet was washed with 70% ethanol and centrifuged for a further 10 min at 14,000× *g*. The pellet was dried for 15 min at 23 °C, re-suspended in 30 µL MQ-H_2_O containing RNAse and then incubated for 1 h at 37 °C. DNA quality and quantity was determined by both agarose gel electrophoresis and by using a Nanodrop spectrophotometer (Thermo Fisher Scientific, Waltham, MA, USA).

### 4.3. Digestion of Plasmid and Genomic DNA with Restriction Enzymes

Restriction enzymes were obtained from Promega UK Ltd. (Southampton, Hampshire, UK) or New England Biolabs (Ipswich, MA, USA). DNA (0.2–1.0 μg) was digested with 5 to 10 units of enzyme in a total volume of 30 µL. For Southern blot analysis, digestion was performed by incubating 50 μg of genomic DNA with 60 units of restriction enzyme in a total volume 50 µL. In both cases, the mixtures were incubated at 37 °C for 16 h.

### 4.4. L. prolificans Genome and Primer Design

The full genome sequence of *L. prolificans* strain 3.1 archived at Biosciences, University of Exeter [38] was used for primer design. Primers were constructed by using the online resource (http://depts.washington.edu/bakerpg/primertemp/primermelttemp.html) and for reverse complements (http://arep.med.harvard.edu/labgc/adnan/projects/Utilities/revcomp.html). The primers used in this study are shown in Table 1.

### 4.5. Polymerase Chain Reaction

Polymerase Chain Reaction (PCR) was used to amplify DNA fragments in an Applied Biosystems GeneAmp^®^ PCR System 2400 cycler (Applied Biosystems, Foster City, CA, USA). GoTaq^®^ Green Master Mix (Applied Biosystems, Foster City, CA, USA), Phusion High Fidelity DNA polymerase (New England Biolabs, Ipswich, MA, USA) or Long PCR enzyme mix (Thermo Fisher Scientific, Waltham, MA, USA) were used according to the manufacturer’s instructions. For GoTaq, the reaction mixture contained 12.5 µL GoTaq Green Master Mix, 10 μM of forward and reverse primer, 50 ng of genomic DNA and nuclease free water to a total volume of 50 µL and PCR cycling conditions were an initial denaturation step at 98 °C for 3 min followed by 35 cycles at 98 °C for 1 min, 53.5–20 °C (depending on annealing temperature of primers) for 1 min and at 72 °C, followed by a final extension step at 72 °C for 10 min. Phusion High Fidelity DNA polymerase reaction mixture contained 5 µL of 5× Phusion HF buffer, 0.5 µL of 10 mM dNTPs, 0.25 µL Phusion, 10 μM of forward and reverse primer, 50 ng of genomic DNA and nuclease-free water to a final volume of 50 µL. PCR cycling conditions were an initial denaturation step at 98 °C for 30 s followed by 35 cycles of 98 °C for 10 s, 62 °C for 30 s and 72 °C for 45 s, with a final extension for 10 min at 72 °C. Long PCR enzyme mix was used for *PKS1* gene amplification. The PCR reaction mixture contained 5 µL of 10× Long PCR buffer with 15 mM MgCl_2_, 1 µL of 10 mM dNTPs, 1 µL of 10 μM of forward and reverse primer, 50 ng of genomic DNA, Long PCR enzyme mix to a total volume of 50 µL with nuclease-free water. PCR cycling conditions were an initial denaturation step at 94 °C for 3 min, 10 cycles at 94 °C for 20 s, 50 °C for 30 s and 68 °C for 7 min, followed by 25 cycles of 94 °C for 20 s, 50 °C for 30 s, 68 °C for 7 min. The final extension step was at 68 °C for 1 min.

### 4.6. Electrophoresis and Purification of Genomic DNA and PCR Products

Agarose gel electrophoresis was used to fractionate digested DNA. The DNA was separated in 0.8% agarose gels and stained with ethidium bromide after mixing with loading dye (50 mL glycerol, 0.25 g bromophenol blue, 5 mL of 0.5 M EDTA (pH 8.0) and 45 mL MQ-H_2_O). Electrophoresis was carried out in 1× Tris-borate EDTA buffer (TBE; 0.09 M Tris-borate, 0.002 M EDTA) for 1 h at 100 V. The DNA fragments were visualized using a UV transilluminator and fragment sizes was determined by comparison with a 1-kb ladder (Promega). Gel images were captured by using an Image Master VDS-CL gel documentation system (GE Healthcare Life Sciences, Little Chalfont, Buckinghampshire, UK) fitted with a Fujifilm FTI-500 Thermal Imaging system (GE Healthcare). DNA fragments were purified from agarose gels using Wizard^®^ SV gel and PCR Clean-Up Systems (Promega) according to the manufacturer’s instructions. Purified DNA was stored at −20 °C until required.

### 4.7. DNA Ligation

Ligation of DNA fragments was carried out using the In-Fusion^®^ HD Cloning kit (Clontech Laboratories Inc., Mountain View, CA, USA) according to the manufacturer’s instructions. The ligation reaction components were combined in a PCR tube in the following order: 2 μL 5× In-Fusion HD enzyme premix, 50 ng linearized vector, 200 ng purified PCR fragment and MQ-H_2_O to a total volume of 10 μL. The cloning reaction was incubated at 50 °C for 15 min and then kept on ice until required.

### 4.8. Transformation of Competent Cells

Stellar competent *Escherichia coli* cells (Clontech) were thawed, placed on ice and mixed gently for even distribution. Fifty-μL of component cells were combined gently with 5 ng (2.5 μL) of In-Fusion cloning reaction. The tubes were incubated on ice for 30 min, heat-shocked at 42 °C for 45 s and then transferred to ice for 1–2 min. SOC medium (2% tryptone, 0.5% yeast extract, 10 mM NaCl, 2.5 mM KCl, 10 mM MgCl_2_, 20 mM glucose) preheated to 37 °C was added to a total volume of 500 μL. The tube was incubated for 1 h at 37 °C with shaking (200 rpm) in an Innova 4000 rotary incubator. One hundred-μL were streaked on LB agar plates (LB broth (tryptone, 10 g/L, yeast extract, 5 g/L, NaCl, 10 g/L, pH 7.5) containing 18 g/L agar) overlaid with X-Gal (40 µg/mL) and supplemented with 1 mL of 100 μg/mL ampicillin. Plates were inverted and incubated at 37 °C for 16 h. Colonies were selected based on white-blue screening.

### 4.9. Plasmid Isolation (Quick-Prep Method)

A single colony of transformed bacteria was used to inoculate 10 mL of LB broth containing 10 µL of 100 μg/mL ampicillin. The culture was incubated overnight at 37 °C with shaking (180 rpm) in an Innova 4000 rotary incubator (Eppendorf). A 1 mL aliquot of culture was transferred to a micro-centrifuge tube and, after centrifugation at 13,000× *g* for 1 min, the supernatant was discarded and the pellet re-suspended in 100 µL lysis solution (250 μL 1 M Tris-HCl (pH8.0), 200 μL 0.5 M EDTA, 170 μL, 60% sucrose, 2.4 mL MQ-H_2_O) and vortexed. Two hundred-μL of alkaline solution (100 µL 10 M NaOH, 500 µL 10% SDS, 4.4 mL MQ-H_2_O) was added and the samples were placed on ice for 10 min with occasional shaking. One hundred and fifty-μL of 3 M sodium acetate (pH 5.2) were added and the samples were incubated on ice for a further 10 min and then centrifuged (13,000× *g*) for 10 min at 4 °C. The supernatant was transferred to a fresh tube containing 1 mL of 100% ethanol and stored on ice for 10 min before centrifugation at 13,000× *g* for 10 min. This process was repeated with 70% ethanol, the pellet dried at 23 °C and then re-suspended in 30 µL of MQ-H_2_O containing 7 µL RNase (10 mg/mL). The DNA was stored at −20 °C until used.

### 4.10. Plasmid DNA Isolation (Midi-Prep Method)

High quality plasmid DNA was isolated using the PureYield™ Plasmid Midiprep System (Promega) according to the manufacturer’s instructions. A single colony of bacterial cells was grown in 50 mL LB containing 1 mL of a 100 μg/mL ampicillin solution for 16 h at 37 °C with shaking (180 rpm) in an Innova 4000 rotary incubator (Eppendorf). The cells were centrifuged at 5,000× *g* for 10 min and the supernatant discarded. The pellet was re-suspended in 3 mL of cell re-suspension solution (50 mM Tris (pH 7.5), 10 mM EDTA, and 100 μg/mL of RNase). Three-mL of cell lysis solution (0.2 M NaOH, 1% (*w*/*v*) SDS) were added and the contents of the tube were mixed by inverting 3–2 times. After 3 min incubation at 23 °C, 5 mL of neutralization solution (4.09 M guanidine hydrochloride, 0.759 M potassium acetate, 2.12 M glacial acetic acid, pH 4.2) was added, the tube was inverted 5–20 times and the lysate centrifuged at 14,000× *g* for 15 min. The PureYield™ purification system was used to purify DNA according to the manufacturer’s instructions and DNA stored at −20 °C until required.

### 4.11. Protoplast Transformation

Spores were harvested from 2-week-old OA cultures, inoculated into 100 mL TCM and incubated for 3 days at 30 °C with shaking (35 rpm) in an Innova 4000 rotary incubator. Hyphal biomass was harvested by filtration through Miracloth and washed and dried as described. The mycelium was transferred to a 50 mL Falcon tube containing 40 mL OM buffer/Glucanex (44 g MgSO_4_·7H_2_O, 1.5 mL 10 mM NaPO_4_, 1.8 g of 5% Glucanex (Novo Nordisk, Copenhagen, Denmark), pH 5.6) and incubated at 30 °C with shaking (75 rpm) for 3 h. The contents of the tube were transferred to sterile polycarbonate Nalgene Oakridge tubes (Thermo Fisher) and the protoplasts were overlaid with chilled ST buffer (0.6 M Sorbitol and 0.1 M Tris-HCl (pH 7.0)). The protoplasts were centrifuged at 5000× *g* for 15 min at 4 °C using a swinging bucket rotor (Beckman JS-13.1, Beckman Coulter Inc., Brea, CA, USA) in a Beckman J2.MC centrifuge (Beckman Coulter). The protoplasts were recovered at the OM/ST interface and transferred to a sterile Oakridge tube, which was filled with cold STC buffer (1.2 M Sorbitol, 10 mM Tris-HCl (pH 7.5), 10 mM CaCl_2_). Protoplasts were pelleted at 3000× *g* for 10 min at 4 °C and washed twice with 10 mL STC, with complete re-suspension each time. Protoplasts were re-suspended in 1 mL STC and numbers quantified using a haemocytometer. Protoplasts were combined with 3 µg DNA in a sterile micro-centrifuge tube in a final volume of 150 µL and incubated for 15 min at 23 °C. One-mL of PTC (60% PEG4000, 10 mM Tris-HCl (pH 7.5), 10 mM CaCl_2_) was added and the contents were mixed by gentle inversion. The protoplasts were incubated at 23 °C for 15 min, added to 150 mL molten (45 °C) OCM agar (osmotically stabilized CM; 50 mL nitrate salt solution, 1 mL trace elements (22 mg/L zinc sulphate heptahydrate, 11 mg/L boric acid, 5 mg/L manganese(II) chloride tetrahydrate, 5 mg/L iron(II) sulphate heptahydrate, 1.7 mg/L cobalt(II) chloride hexahydrate, 1.6 mg/L copper(II) sulphate pentahydrate, 1.5 mg/L sodium molybdate dehydrate, 50 mg/L EDTA), 10 g glucose, 2 g peptone, 1 g yeast extract, 1 g casamino acids, 1 mL vitamin solution (0.001 g/L, biotin, 0.001 g/L, pyridoxine, 0.001 g/L, thiamine, 0.001 g/L riboflavin, 0.001 g/L, 0.001 g/L nicotinic acid), 273.84 g sucrose and 15 g agar in a final volume of 1 L, adjusted to pH 6.5 with 1 M NaOH) and the protoplast suspension poured into sterile 9 cm plastic culture dishes for incubation in the dark at 30 °C for 16 h. The plates were then overlaid with complete medium (CM; 10 g/L glucose, 2 g/L, peptone, 1 g/L, yeast extract (BD Biosciences UK, Oxford, Oxfordshire, UK), 1 g/L casamino acids, 0.1% trace elements, 0.1%, 0.1% vitamin solution, 6 g/L NaNO_3_, 0.5 g/L, KCl, 0.5 g/L, MgSO_4_, 1.5 g/L, KH_2_PO_4_, (pH to 6.5 with NaOH), 15 g/L agar) containing 600 µg/mL hygromycin B (Calbiochem) and were then incubated for 1 week in the dark at 30 °C. Colonies resistant to hygromycin B were sub-cultured on CM containing 200 µg/mL hygromycin B and finally onto OA. For selection of sulfonylurea-resistant transformants, CM was replaced with BDCM medium (yeast nitrogen base without amino acids and ammonium sulfate, agar 1.7 g/L, ammonium nitrate, 2 g/L, asparagine, 1 g/L, glucose, 10 g/L, sucrose, 0.8 M, pH 6.0) with chlorimuron ethyl at a concentration of 300 μg/mL in the overlay, chlorimuron ethyl at a concentration of 100 μg/mL in BDCM sub-cultures and finally growth on OA.

### 4.12. Southern Blotting

Southern blotting was carried out according to the protocol of Southern [57]. Thirty-µg of genomic DNA was digested overnight using an appropriate restriction enzyme. The products of digestion were separated on 0.8% agarose gels at 100 V for 3 h. Gels were immersed in 0.25 M HCl for 15 min, followed by 0.4 M NaOH for a further 15 min, for de-purination and neutralization respectively. Gel blots were carried out by placing gels onto Whatman 3 mm paper wetted with 0.4 M NaOH and supported by a Perspex sheet with the ends of the paper immersed in 0.4 M NaOH. Gels were covered with Hybond-NX membrane (GE Healthcare Life Sciences), two layers of Whatman 3 mm paper and paper towel (Kimberley Clark). Finally, a 500 g weight was placed on the blotted gel and the blot was incubated for 16 h at 23 °C. Transferred DNA was UV-cross linked to the membrane using a BioLink BLX crosslinker (Sigma-Aldrich).

DNA probes were amplified by using Phusion HF buffer and the appropriate primers shown in Table 1 and reaction mixtures labelled with PCR DIG labelling mix (Sigma-Aldrich). Membranes were incubated in Hybaid hybridization bottles (Thermo Fisher) in a hybridization oven (Thermo Fisher) with Southern hybridization buffer (500 mL 1 M NaPO_4_ (pH 7.0), 350 mL 20% SDS and Seradest added to a final volume of 1 L) at 62 °C for 30 min. Probes were denatured by boiling for 10 min and then added to hybridization bottles containing the membranes. Following hybridization at 62 °C for 16 h, the membranes were washed twice (15 min each) at 62 °C with Southern wash buffer (100 mL 1 M NaPO_4_ (pH 7.0), 50 mL 20% SDS and MQ-H_2_O to 1 L) and once at 23 °C using 20 mL DIG wash buffer 1 (6 mL Tween-20, 1.994 mL DIG buffer (maleic acid 0.1 M, NaCl 0.15 M, adjusted to pH 7.5 with 5 M NaOH) and with MQ-H_2_O added to 2 L) for 5 min. Membranes were submerged in 50 mL of blocking buffer (1 g semi-skimmed milk powder and 100 mL DIG buffer 1) for 30 min at 23 °C and then incubated with 50 mL of antibody solution (2.5 µL anti-digoxigenin-AP Fab fragments (Sigma-Aldrich) and 50 mL blocking buffer) under the same conditions. After two 15 min washes with DIG wash buffer and equilibriation with 20 mL DIG buffer 3 (200 mL of 1 M Tris-HCl, 40 mL of 5 M NaCl, 100 mL of 1 M MgCl_2_ (pH 9.5) with MQ-H_2_O added to 2 L) for 5 min, membranes were incubated for 5 min with 2 mL CDP-star^®^ chemiluminescent substrate solution (Sigma-Aldrich) and dried completely. Finally, the membrane was exposed to X-ray film (Fuji Photo Film (UK) Ltd., Manchester, Cheshire, UK) placed in a film cassette and incubated at 37 °C for 15 min. Films were developed using an OPTIMA X-Ray Film Processor (Protec GmbH, Oberstenfield, Germany).

### 4.13. Hyphal Growth, Sporulation and Spore Pigmentation

Strains were inoculated centrally onto replicate OA plates and colony diameters were measured over a 2-week growth period at 30 °C, with spore production quantified after 14 day. Spores were suspended in 20 mL dH_2_O using plastic L-shaped spreaders, filtered through Miracloth, and spore concentrations determined using a haemocytometer. There were 3 replicates for each experiment and experiments were repeated 3 times.

### 4.14. Oxidative Killing by H_2_O_2_

Preliminary investigations set out to establish the molarity and exposure time to H_2_O_2_ that resulted in a 50% reduction in survival of the wild-type strain 3.1. Replicate spore suspensions with a concentration of 10^3^ spores/mL were suspended in 100 mM phosphate buffer (pH 7.0) comprising a range of mM concentrations of H_2_O_2_. Following exposure, 300 µL of spore suspensions were spread on the surface of SDA plates and incubated for 3 day at 30 °C in the dark. The control consisted of untreated spores (suspended in buffer only) and percentage survival was determined from the numbers of colonies that developed from treated spores compared to the untreated control. Through this process it was determined that exposure of spores to 160 mM H_2_O_2_ for 60 min resulted in 50% survival of treated spores of the wild-type compared to the matched untreated control. All subsequent experiments using mutant strains were conducted using this same treatment regime and percentage survival relative to matched controls determined as described. There were 3 replicates for each strain and experiments were conducted three times.

### 4.15. Sensitivity to UV Radiation

Preliminary investigations aimed to establish the UV dose at 254 nm that resulted in a 50% reduction in survival of the wild-type strain 3.1. Replicate spore suspensions with a concentration of 10^3^ spores/mL were exposed to UV light generated by a HL-2000 HybriLinker Hybridization oven (UVP) with doses of 100, 200, 300, 400 and 500 mJ/cm^2^. Following exposure, 300 µL of spore suspensions were spread on the surface of SDA plates and incubated for 3 days at 30 °C in the dark. The control consisted of non-irradiated spores and percentage survival was determined from the numbers of colonies that developed from irradiated spores compared to the non-irradiated control. Through this process it was determined that a UV dose of 200 mJ/cm^2^ resulted in 50% survival of irradiated spores of the wild-type compared to the matched non-irradiated control. All subsequent experiments using mutant strains were conducted at this dose and percentage survivals relative to matched controls determined as described. There were 3 replicates for each strain and experiments were conducted three times.

### 4.16. Sensitivity to Amphotericin B

The sensitivities of the mutants and strain 3.1 to the polyene amphotericin B (AmB; Sigma, A2942) were tested using a liquid culture method. Tissue culture medium was amended with a liquid formulation of AmB (Sigma; A2942) to give final concentration of 32 µg/mL and the control consisted of TCM amended with an equivalent volume of sterile MQ-H_2_O only. Tissue culture flasks (75 cm^2^) containing AmB or control media were inoculated with spores of the *L. prolificans* strains, or the drug-sensitive pathogen *Aspergillus fumigatus* (strain AF293), to give a final concentration of 10^6^ spores/mL. Flasks were incubated for 3 days at 30 °C with shaking (48 rpm) in an Innova 4000 rotary incubator and the mycelium was harvested, dried at 70 °C for 48 h and dry weights obtained. The experiment was repeated three times with three replicates for each strain and treatment.

### 4.17. Statistical Analysis

Unless otherwise stated, numerical data were analyzed using the statistical program Minitab (Minitab 16, Minitab^®^, Coventry, UK). Analysis of variance (ANOVA) was used to compare means of more than two data sets and Post-hoc Tukey-Kramer analysis was then performed to distinguish which sets were significantly different from one another. For comparisons of percentages, data was transformed using the arcsin^−1^ function prior to statistical analysis.

## Figures and Tables

**Table 1:** Details of primer sequences used in this study.

Primer Name	Sequence 5′-3′	Product
Lpscd1-F	GATCGCCATCCCAGCCATCA	*Lpscd1* ORF
Lpscd1-R	ACGTGGCAAGGGTTGGATCC	*Lpscd1* ORF
Lpscd1-LFF	CGTCAGCTTTGGAAAACAAC	*Lpscd1* LF
Lpscd1-LFR	GTCGTGACTGGGAAAACCCTGGCGACGGTAGCCATGTTTTCCGA	*Lpscd1* LF
Lpscd1-RFF	TCCTGTGTGAAATTGTTATCCGCTTATCTGGCGTCAATCGGAAA	*Lpscd1* RF
Lpscd1-RFR	ACATGTTGTTTTGCACGCTT	*Lpscd1* RF
HY split	GGATGCCTCCGCTCGAAGTA	*Lpscd1* LF+HY
YG split	CGTTGCAAGACCTGCCTGAA	*Lpscd1* YG+RF
M13F	CGCCAGGGTTTTCCCAGTCACGAC	-
M13R	AGCGGATAACAATTTCACACAGGA	-
Lppks1-F	TCTCGGTTTCTCCATGCAAA	*Lppks1* ORF
Lppks1-R	CCTGCACAAACAATTCGTTA	*Lppks1* ORF
Lppks1-LFF	AATCAAGTCGCCAGGACCTT	*Lppks1* LF
Lppks1-LFR	GTCGTGACTGGGAAAACCCTGGCGTTTGCATGGAGAAACCGAG	*Lppks1* LF
Lppks1-RFF	TCCTGTGTGAAATTGTTATCCGCTGAGCTGTATCACTGAGACA	*Lppks1* RF
Lppks1-RFR	GAGGCCATTCAAAGATCCCA	*Lppks1* RF
Lppks1-f1	TAGAACTAGTGGATCCGTCTCACATCGTCGTACTATAAT	*Lppks1* gene fragment
Lppks1-r1	CGGTATCGATAAGCTCGGCACTAGGATATAAACCCTCTT	*Lppks1* gene fragment
Lppks1-ComF	GAGTTCTGCCGTCAGGGAAA	*Lppks1* probe
Lppks1-ComR	GAAGATGTGGCGCATGGTAG	*Lppks1* probe

The reverse complement of M13 forward and reverse sequences, used with HY split and YG split respectively, are shown underlined. ORF: open reading frame; LF: left flank; RF: right flank; LFF: left flank forward; LFR: left flask reverse; RFF: right flank forward; RFR: right flank reverse.
